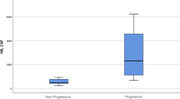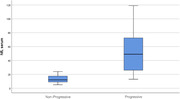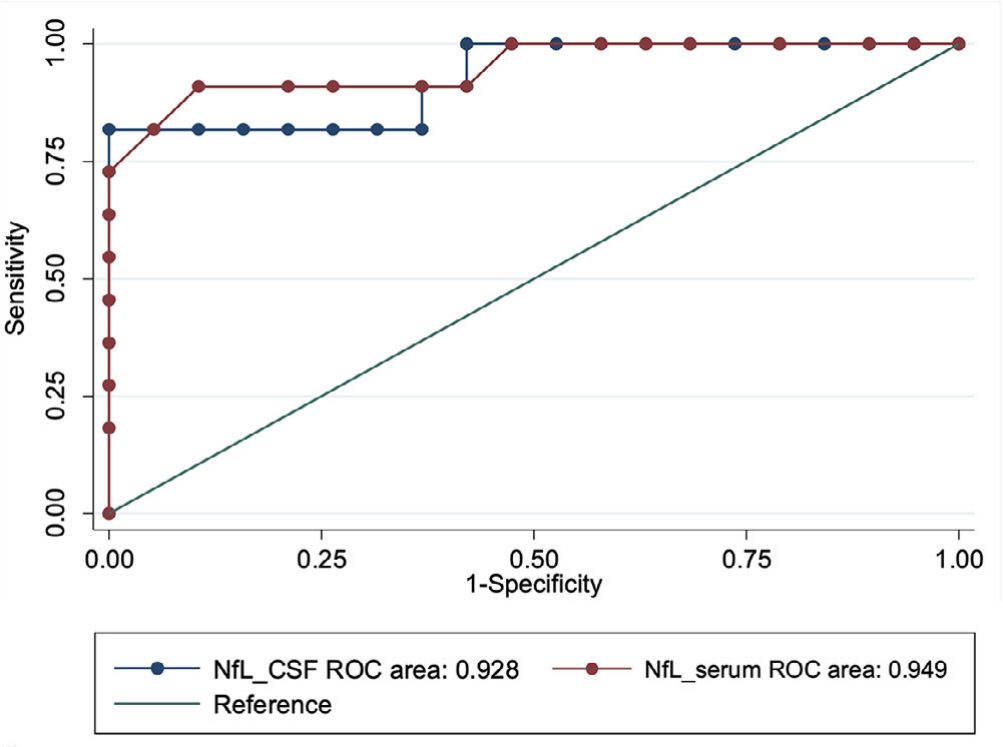# The role of neurofilaments light chain in discriminating behavioral variant frontotemporal dementia from non‐neurodegenerative mimics

**DOI:** 10.1002/alz.089379

**Published:** 2025-01-09

**Authors:** Chiara Gallingani, Giulia Vinceti, Chiara Carbone, Manuela Tondelli, Davide Salvatori, Simone Salemme, Roberta Bedin, Teresa Urbano, Ludovico Luchetti, Giordano Gentile, Marco Battaglini, Annalisa Chiari, Giovanna Zamboni

**Affiliations:** ^1^ Università di Modena e Reggio Emilia, Modena Italy; ^2^ Neurologia, Azienda Ospedaliero Universitaria di Modena, Modena Italy; ^3^ Scuola Internazionale di Studi Avanzati, Università di Camerino, Camerino Italy; ^4^ CREAGEN ‐ Centro di Ricerca in Epidemiologia Ambientale, Genetica e Nutrizionale, Università di Modena e Reggio Emilia, Modena Italy; ^5^ Università di Siena, Siena Italy; ^6^ SIENA Imaging SRL, Siena Italy

## Abstract

**Background:**

The measurement of serum and cerebrospinal fluid (CSF) neurofilaments light chain (NfLs) has been proven promising in differentiating the behavioral variant frontotemporal dementia (bvFTD) from non‐neurodegenerative mimics, including primary psychiatric disorders and non‐progressive cognitive/behavioral changes. However, studies on this topic are based on clinical diagnosis, which remains challenging and potentially confounded by the overlapping clinical phenotypes. We investigated the role of NfLs in this field by classifying patients based on the presence/absence of pathological longitudinal brain volume changes.

**Method:**

We prospectively recruited subjects with early‐onset (< 65 yo) mild cognitive/behavioral impairment suggestive of potential bvFTD. At baseline they underwent neuropsychological assessment, magnetic resonance imaging (MRI) brain imaging, and CSF and serum collection for NfLs measurement. They were followed‐up clinically and repeated the MRI scan at 18 months. Single‐subject yearly percentage brain volume change (PBVC/y) was calculated using the FMRIB Software Library tool SIENA and compared to normative values. Subjects with a PBVC/y exceeding the 95^th^ normative percentile were classified as *Progressive*, in line with a bvFTD diagnosis. Otherwise, they were indicated as *Non‐Progressive*, suggestive of a non‐neurodegenerative condition. Differences in NfLs levels between *Progressive* and *Non‐Progressive* individuals and their predictive value were evaluated through non‐parametric tests and univariate logistic regression models.

**Result:**

30 patients were recruited: 19 were classified as Non‐Progressive and 11 as Progressive. Both CSF and serum NfLs values were significantly higher in patients with atrophy progression compared to the stable ones (2992,27 vs 558,53 pg/dl, p < 0.001; 55,91 vs 13,47 pg/dl, p < 0.001). Higher serum NfLs levels at baseline were predictors of atrophy progression (p = 0.041, OR = 1.301), whereas only a trend of significance was seen for CSF NfLs (p = 0.055, OR = 1.006). A cut‐off of 21.5 pg/dl for serum NfLs differentiated the two groups with 81.82% sensitivity and 94.74% specificity (AUC = 0.949); a cut‐off of 972 pg/dl for CSF NfLs yielded discriminative sensitivity of 81.82% and specificity of 100% (AUC = 0.928).

**Conclusion:**

We confirmed that NfLs measurement in biological fluids at baseline is a reliable biomarker to discriminate between bvFTD patients and non‐neurodegenerative mimics, objectively distinguished based on longitudinal atrophy progression.